# Parallel evolution of two distinct lymphoid proliferations in clonal haematopoiesis

**DOI:** 10.1111/his.14619

**Published:** 2022-03-01

**Authors:** Ayoma D Attygalle, Rachel Dobson, Pui Kwan Chak, Katherine M Vroobel, Dorte Wren, Hood Mugalaasi, Yvonne Morgan, Manmit Kaur, Raida Ahmad, Zi Chen, Kikkeri N Naresh, Ming‐Qing Du

**Affiliations:** ^1^ Department of Histopathology The Royal Marsden Hospital London UK; ^2^ Division of Cellular and Molecular Pathology, Department of Pathology University of Cambridge Cambridge UK; ^3^ Department of Anatomical and Cellular Pathology Prince of Wales Hospital Shatin New Territories Hong Kong; ^4^ Genomic Diagnostics Laboratory, Manchester Centre for Genomic Medicine Manchester University NHS Foundation Trust, Saint Mary's Hospital Manchester UK; ^5^ Clinical Genomics, Haematological Diagnostic Malignancy Service The Royal Marsden Hospital Sutton UK; ^6^ Department of Haematology Luton & Dunstable University Hospital Luton UK; ^7^ Department of Cellular Pathology Imperial College Healthcare NHS Trust, Charing Cross Hospital London UK; ^8^ Centre for Haematology Imperial College London London UK; ^9^ Clinical Research Division Fred Hutchison Cancer Research Center Seattle WA USA

**Keywords:** angioimmunoblastic T‐cell lymphoma, clonal haematopoiesis, secondary lymphoid neoplasm, *TET2* and *DNMT3A* mutation

## Abstract

**Aims:**

Angioimmunoblastic T‐cell lymphoma (AITL) is genetically characterized by *TET2* and *DNMT3A* mutations occurring in haematopoietic progenitor cells, and late events (e.g. the *RHOA*‐G17V mutation) associated with malignant transformation. As *TET2*/*DNMT3A*‐mutated progenitor cells can differentiate into multilineage progenies and give rise to both AITL and myeloid neoplasms, they may also have the potential to lead to other metachronous/synchronous neoplasms. We report two cases showing parallel evolution of two distinct potentially neoplastic lymphoid proliferations from a common mutated haematopoietic progenitor cell population.

**Methods and results:**

Both cases presented with generalized lymphadenopathy. In case 1 (a 67‐year‐old female), an initial lymph node (LN) biopsy was dismissed as reactive, but a repeat biopsy showed a nodal marginal zone lymphoma (NMZL)‐like proliferation with an increase in the number of T‐follicular helper (TFH) cells. Immunohistochemistry, and clonality and mutational analyses by targeted sequencing of both whole tissue sections and microdissected NMZL‐like lesions, demonstrated a clonal B‐cell proliferation that harboured the *BRAF*‐G469R mutation and shared *TET2* and *DNMT3A* mutations with an underlying *RHOA‐*G17V‐mutant TFH proliferation. Review of the original LN biopsy showed histological and immunophenotypic features of AITL. In case 2 (a 66‐year‐old male), cytotoxic T‐cell lymphoma with an increase in the number of Epstein–Barr virus‐positive large B cells was diagnosed on initial biopsy. On review together with the relapsed biopsy, we identified an additional occult neoplastic TFH proliferation/smouldering AITL. Both T‐cell proliferations shared *TET2* and *DNMT3A* mutations while *RHOA*‐G17V was confined to the smouldering AITL.

**Conclusions:**

In addition to demonstrating diagnostic challenges, these cases expand the potential of clonal haematopoiesis in the development of different lineage neoplastic proliferations.

## Graphical abstract

Two unique cases show parallel evolution of distinct lymphoid neoplasms from a common *TET2/DNMT3A* mutated haematopoietic progenitor. In case 1, an EBV‐negative clonal B‐cell proliferation mimicking nodal marginal zone B‐cell lymphoma (NMZL) obscured an underlying angioimmunoblastic T cell lymphoma (AITL). In case 2, a predominant CD8+ cytotoxic T‐cell proliferation/lymphoma concealed an occult neoplastic T‐helper cell proliferation/smouldering AITL.

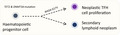

## Introduction

Angioimmunoblastic T‐cell lymphoma (AITL) is the commonest non‐cutaneous peripheral T‐cell lymphoma (TCL) in western Europe.^1^ AITL and other nodal lymphomas of T‐follicular helper (TFH) cell origin (TCLs‐TFH) were recently grouped as an umbrella category in the 2016 World Health Organization classification.^2^ These lymphomas are typically aggressive, with a 5‐year overall survival of 34%,^1^ although there are occasional long‐term survivors.^3^ The mutational landscape of AITL/TCL‐TFH has been well characterized, with frequent early occurrence of *TET2* and *DNMT3A* mutations in haematopoietic stem cells, and *RHOA*‐G17 and *IDH2*‐R172 mutations as later pathogenic events in tumour cells only.^4^, ^5^

*TET2* and *DNMT3A* mutations are commonly found in clonal haematopoiesis and myeloid neoplasms.^6^, ^7^, ^8^ These mutated haematopoietic stem cells can differentiate into multilineage progenies, and may give rise to both AITL/TCL‐TFH and myeloid neoplasms in the same patients after the acquisition of additional distinct genetic changes.^9^, ^10^ In patients with AITL/TCL‐TFH, the *TET2* and *DNMT3A* mutations detected in neoplastic TFH cells are also seen in non‐neoplastic B and CD8+ T cells.^4^, ^11^, ^12^, ^13^ It is unclear whether these non‐neoplastic B and T cells with *TET2* and *DNMT3A* mutations may have growth or survival advantages for clonal expansion, particularly in view of the potential stimulation by inflammatory responses, and putative T‐cell help from the neoplastic TFH cells.^14^, ^15^ In fact, both minor clonal B‐cell and T‐cell populations are frequently observed alongside the neoplastic TFH cell clone in lymph node (LN) biopsies of patients with AITL, with the former thought to be largely due to Epstein–Barr virus (EBV)‐infected B cells.^16^, ^17^ However, these cell populations are commonly intermingled with the neoplastic TFH cells, creating huge difficulties for their in‐depth characterization, particularly in the absence of fresh tissue for flow cytometry (FCM) and cell sorting analysis.

We have encountered two unique cases in which a lymphoid neoplasm was identified and shown to share the *TET2* and *DNMT3A* mutations seen in an underlying neoplastic TFH cell proliferation/smouldering AITL in each case. In case 1, an EBV‐negative clonal B‐cell proliferation mimicking nodal marginal zone lymphoma (NMZL) obscured an underlying AITL. In case 2, a predominant CD8+ cytotoxic T‐cell (CTC) proliferation/lymphoma concealed an occult neoplastic T‐helper cell proliferation/smouldering AITL.

## Materials and methods

### HISTOPATHOLOGY AND IMMUNOHISTOCHEMISTRY

Local ethical guidelines were followed for the use of tissues for research, with ethical approval (05‐Q1604‐10).

The two cases described were received for consultation by one of the authors (A.D.A.) and reviewed by authors (case 1 by P.K.C., and case 2 by K.N.N., K.M.V., and R.A.). Immunohistochemistry was routinely performed on formalin‐fixed paraffin‐embedded (FFPE) tissue sections (Table S1), and, where indicated, double immunohistochemistry was performed.

### MICRODISSECTION AND DNA EXTRACTION

DNA samples were prepared from whole tissue sections or microdissected tumour areas from FFPE tissue biopsies (Appendix S1). The quality of DNA samples was assessed by polymerase chain reaction (PCR) of variably sized genomic fragments.^18^

### CLONALITY ANALYSIS

The rearranged antigen receptor genes were routinely amplified in duplicate by use of the BIOMED‐2 assays, and analysed with the genescan protocol.^19^

### MUTATION ANALYSES BY TARGETED SEQUENCING

The initial mutation discovery was made by targeted sequencing of the Royal Marsden Hospital (RMH) lymphoma panel (Table S2; Appendix S1), which was used in the clinical diagnostic service. Briefly, purified DNA (1 μg) was subjected to target enrichment with DNA baits (Roche Nimblegen, Switzerland), library preparation with the KAPA HyperPlus Kit (Kapa Biosystems, Wilmington, DE, USA), and finally sequenced with the NovaSeq 6000 system (Illumina, San Diego, CA, USA). The sequence data were assessed for quality and coverage, and variants were called using gatk, mutect2, and pindel.

The variants detected were validated, and further investigated in microdissected cells and other related samples by Fluidigm PCR (Fluidigm Access Array System, South San Francisco, CA, USA) and Illumina MiSeq,^16^ (Illumina, San Diego, CA, USA) or PCR and Sanger sequencing (Appendix S1).

### *RHOA* MUTATION ANALYSIS WITH QUANTITATIVE PCR (qPCR)

The *RHOA*‐G17V mutation was additionally investigated by qPCR with a peptide nucleic acid clamp and a locked nucleic acid probe,^20^ which is highly sensitive, and suitable for use with crude DNA extracts. The qPCR was carried out in triplicate using 2–7.5 μl of crude DNA or 25 ng of purified DNA, with denaturation at 95°C for 30 s, followed by 45 cycles at 95°C for 3 s, and 62°C for 30 s.

## Results

### CASE 1: AITL WITH A SECONDARY EBV‐NEGATIVE CLONAL NMZL‐LIKE PROLIFERATION

A 67‐year‐old female presented with generalized lymphadenopathy and bilateral pleural effusions, but lacked B‐symptoms. Positron emission tomography (PET)–computed tomography (CT) confirmed small‐volume lymphadenopathy above and below the diaphragm (standard uptake value[SUV] maximum of 3.3). A groin LN was excised and reported locally as reactive.

**Figure 1:**
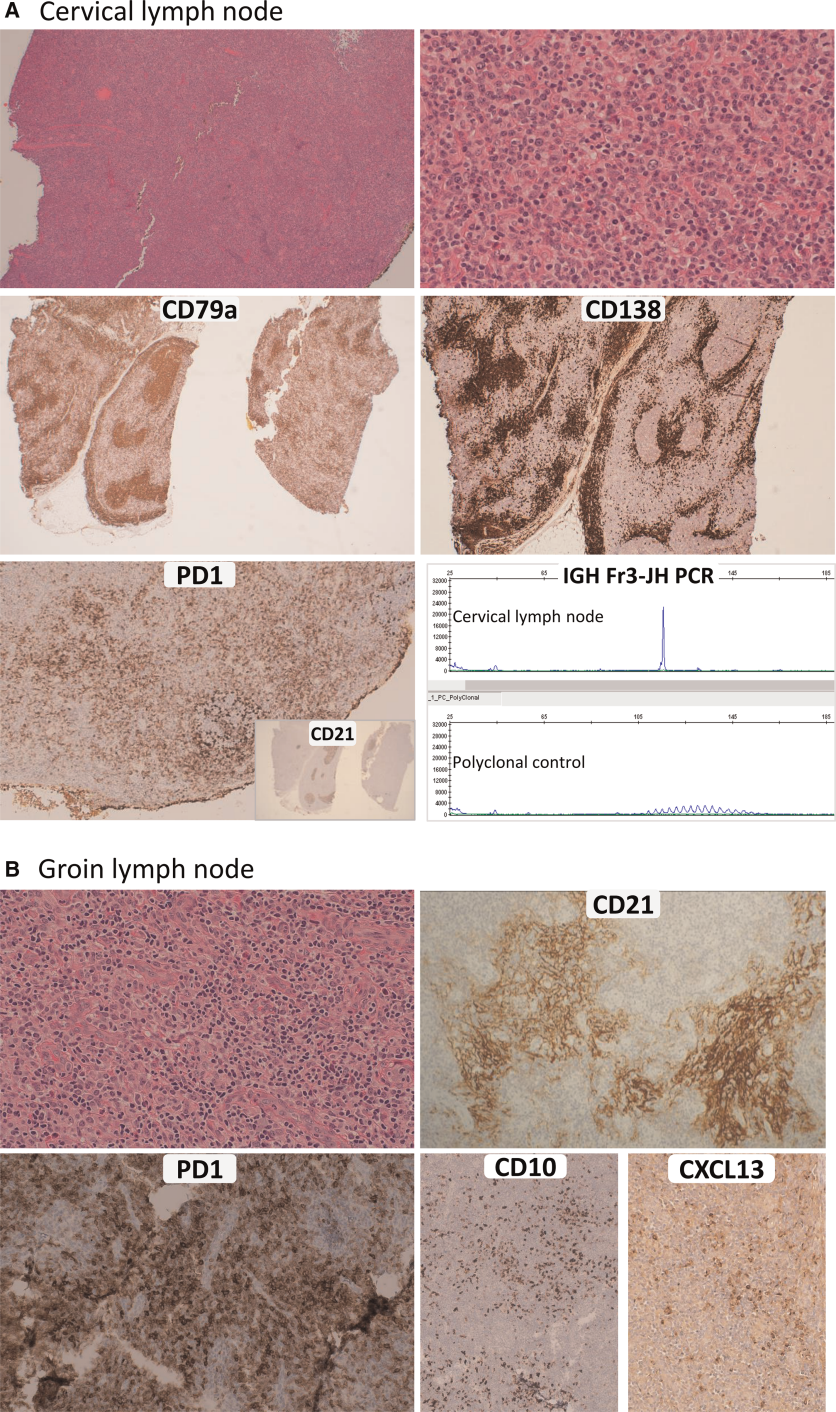
Histopathological and immunohistochemical investigations of lymph node (LN) biopsies in case 1. **A,** A cervical LN showing a florid B‐cell and plasmacytic infiltrate mimicking nodal marginal zone lymphoma (NMZL)/secondary NMZL, obscuring underlying angioimmunoblastic T‐cell lymphoma (AITL). Upper panels: on an haematoxylin and eosin (H&E)‐stained section (left), there is effacement of the nodal architecture by an expanded perifollicular infiltrate (right) composed of small and medium‐sized lymphocytes and mature plasma cells. Middle panels: CD79a staining highlights the B cells and plasma cells (left), whereas CD138 marks the dense clusters of mature plasma cells (right). Lower panels: there is an increase in the number of programmed cell death protein 1 (PD1)‐positive T cells within the germinal centres (strong staining) and also in the perifollicular areas (left), but CD21 staining does not show any extrafollicular follicular dendritic cell (FDC) expansion (inset); a clonal B‐cell population in a cervical LN biopsy was detected by polymerase chain reaction with the BIOMED‐2 assay (IGH FR3‐JH) (right). **B,** A groin LN showing typical features of AITL. Upper panels: on an H&E‐stained section (left) there is striking high endothelial venule (HEV) hyperplasia, amidst which is an infiltrate of medium‐sized atypical lymphoid cells in a background of histiocytes, plasma cells, and eosinophils. On a CD21‐stained section (right), there is prominent FDC expansion, seen to encircle HEVs. Lower panels: the atypical lymphoid cells are strongly positive for PD1 (left), and a proportion are positive for CD10 (left side of the right panel) and CXCL13 (right side of the right panel).

Ten months later, because of persistent lymphadenopathy and effusions, a cervical LN was excised that showed partial effacement by a perifollicular infiltrate of small lymphocytes, scattered intermediate to large cells, and densely clustered mature plasma cells (Figure 1A). Mild high endothelial venule (HEV) hyperplasia was noted. The lymphocytes were mainly CD20+ small B cells. The B cells and plasma cells showed kappa light chain restriction and were EBV‐encoded small RNA (EBER)‐negative by *in‐situ* hybridisation. There was an increase in the number of CD4+/programmed cell death protein 1 (PD1)+/ICOS+/CXCL13+/CD10+ (subset) small to medium‐sized TFH cells (see Table 1 and Figure 1A for a detailed immunoprofile).

**Table 1:** Summary of histopathological and immunophenotypic findings

Case	Site	Cell type	Positive markers	Negative markers	Light chain restriction
Case 1	Cervical LN	Small B cells	CD20, CD79a, PAX5, CD19	CD10 (verified by PAX5/CD10 double staining), bcl‐6, cyclin D1, IgD, CD5, CD23	Kappa
		Mature plasma cells	CD138, CD79a, CD19	CD20, PAX5, cyclin D1, CD56, EBER‐ISH	Kappa
		TFH cells	CD2, CD3, CD5, CD7, CD4, PD1, CD10 (subset), ICOS, CXCL13	CD8	NA
		FDC meshworks	No expansion		
	Groin LN	Small B cells (confined to residual follicles)	CD20, CD79a, PAX5, IgD	EBER	Not done
		Plasma cells	NA (low numbers)	NA (low numbers)	NA
		TFH cells	CD2, CD3, CD5, CD7, CD4, PD1, CD10 (subset), ICOS, CXCL13	CD8	NA
		FDC meshworks	Extrafollicular FDC (CD21+) expansion encircling HEVs		
Case 2	Mediastinal and groin LN	Cytotoxic T cells	CD2, CD3, CD8, TCR‐β, TIA1, granzyme B, CD57 (minority)	CD5, CD7, CD4, PD1, CD10, ICOS, CXCL13 (verified by CD8/PD1, CD8/10 and CD8/CD4 double immunostaining)	NA
		TFH cells (minor population)	CD2, CD3, CD5, CD7, CD4, PD1, CD10, ICOS, CXCL13	CD8	NA
		B cells (variable in size with a moderate large‐cell component)	CD20 (majority), CD79a, EBER‐ISH (in larger cells)	–	Polytypic
		FDC meshworks	Mild FDC (CD21+) expansion around HEVs		
	Bone marrow	Flow cytometry analysis revealed CD3+/CD8+ T cells (45% of all T cells), and additional sCD3–/CD4+/CD10+ T cells (0.75% of all CD45+ cells)			
		Trephine biopsy showed cytotoxic T cells, and an additional minor population of CD4+/ICOS+/PD1 (moderate to strong) + T cells			
	Skin	Subtle dermal infiltrates of neoplastic cytotoxic T cells, and CD4+/PD1 (strong)+/ICOS+/CD10+ TFH cells			

EBER‐ISH, Epstein–Barr virus‐encoded small RNA *in‐situ* hybridisation; FDC, follicular dendritic cell; HEV, high endothelial venule; LN, lymph node; NA, not applicable; PD1, programmed cell death protein 1; TCR, T‐cell receptor; TFH, T‐follicular helper.

Clonality analysis detected a strong B‐cell clone (Figure 1A), but was unsuccessful for T cells, owing to poor DNA quality. Given the relatively indolent clinical presentation, a provisional diagnosis of NMZL was made.

**Figure 2:**
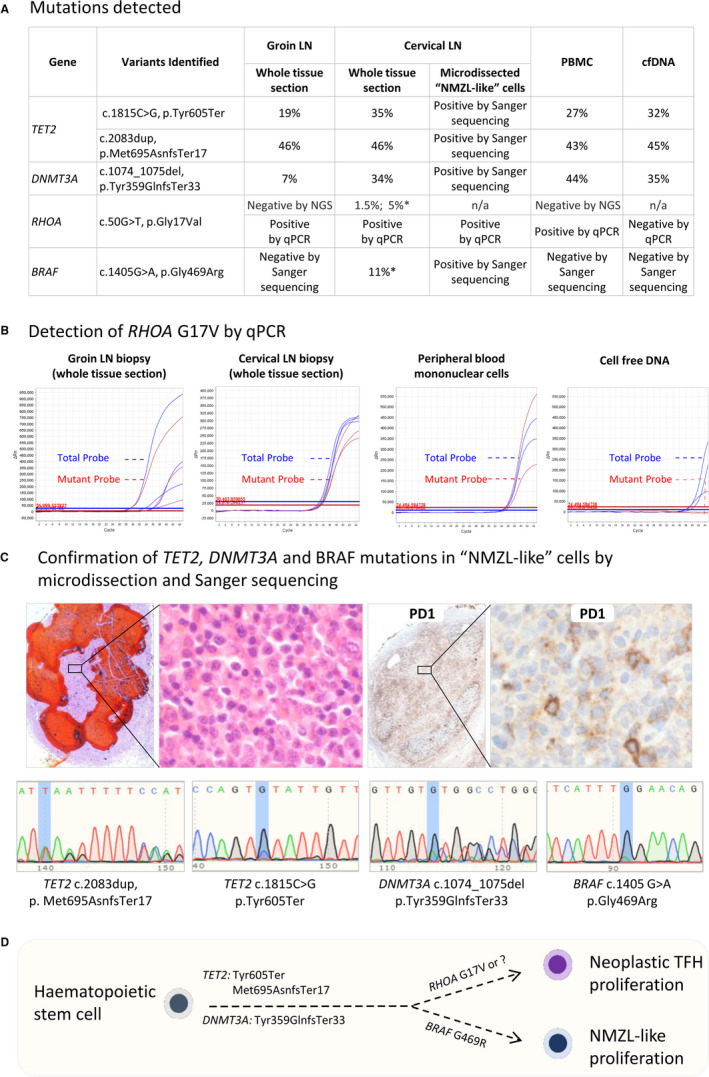
Genetic findings in case 1. **A,** A summary of the mutations detected in various lymph node (LN) biopsies and blood samples by the use of targeted next‐generation sequencing and quantitative polymerase chain reaction (PCR) (qPCR). The allelic frequencies of the variants included are based on Fluidigm PCR and MiSeq sequencing unless otherwise indicated. *Based on Royal Marsden Hospital (RMH) panel sequencing. The sequencing coverage for all the variant loci by the Fluidigm PCR and MiSeq approach is adequate (mean of 976 reads and range of 118–1617 reads passing sequence quality checks), as PCR primers are designed to amplify short sequence fragments. cfDNA, cell‐free DNA; PBMC, peripheral blood mononuclear cell. **B,** Examples of *RHOA*‐G17V mutation analysis by qPCR. The qPCR analysis of whole tissue sections of the groin LN was based on crude DNA preparations with varying amounts of DNA for different replicates, thus giving rise to different profiles, albeit they were all positive. The qPCR analysis of other samples shown in this panel was based on purified DNA. **C,** Confirmation of *TET2*, *DNMT3A* and *BRAF* mutations in ‘nodal marginal zone lymphoma (NMZL)‐like’ B cells by microdissection and Sanger sequencing. The prominent mutant sequence traces indicate that these mutations were present in the clonal NMZL‐like B cells. The areas enriched by the B‐cell/plasmacytic NMZL‐like infiltrate but lacking prominent programmed cell death protein 1‐positive cells were first identified (marked by red line), and used to guide microdissection on consecutive tissue sections. **D,** Parallel evolution of a neoplastic T‐follicular helper cell proliferation/underlying AITL and an NMZL‐like proliferation from a common haematopoietic progenitor cell population that harbour *TET2* and *DNMT3A* mutations.

However, in view of the increase in the number of TFH cells, next‐generation sequencing (NGS) of a lymphoma panel was undertaken to exclude underlying AITL/TCL‐TFH. The sequencing coverage was suboptimal, but nonetheless identified two *TET2* variants, one *DNMT3A* variant, the *RHOA‐*G17V variant, and the *BRAF* G469R variant. There was no evidence of mutations in *IDH2* and *PLCG2*, or other B‐cell lymphoma genes, including *CARD11*, *CCND3*, *CREBBP*, *EP300*, *EZH2*, *FOXO1*, *MAP2K1*, *MEF2B*, and *MYD88*. The above mutations were confirmed by Fluidigm PCR/Illumina MiSeq, PCR/Sanger sequencing and *RHOA*‐G17V qPCR (Figure 2A; Figure S1). *TET2* and *DNMT3A* mutations were detected in both peripheral blood mononuclear cells (PBMCs) and cell‐free DNA (cfDNA), whereas the *RHOA*‐G17V mutation was found only in PBMCs, bu not in cfDNA (Figure 2A,B).

The previous groin LN biopsy was reviewed, and showed typical features of AITL, with extrafollicular follicular dendritic cell (FDC) hyperplasia and small to medium‐sized TFH cells concentrated around hyperplastic HEVs (Figure 1B; Table 1), but no significant B‐cell infiltrate. Clonality analyses failed because of poor DNA quality. Targeted sequencing detected identical *TET2* and *DNMT3A* mutations to those seen in the cervical LN (Figure 2A). The *RHOA*‐G17V mutation was detected by qPCR (Figure 2B), but the results of targeted NGS were inconclusive, since it was detected in only one of the two replicates, most likely because of a low variant allele frequency (VAF). PCR and Sanger sequencing analysis of DNA samples from areas enriched for TFH cells by microdissection failed to detect the *RHOA*‐G17V mutation, further indicating that the mutation had a low allele frequency below the detection limit of the Sanger method. Despite the unusual indolent clinical course, a revised diagnosis of AITL was made, with the NMZL‐like clonal B‐cell and plasmacytic proliferation in the cervical LN being interpreted as a synchronous event.

To investigate whether the aforementioned mutations were present in the clonal B cells in the cervical LN, we performed Sanger sequencing on DNA samples extracted from two small microdissected tissue areas (~2500 cells) rich in the B‐cell/plasmacytic NMZL‐like infiltrate (Figure 2C). The analyses identified the *TET2*, *DNMT3A* and *BRAF* mutations, with a strong mutant sequence trace indicating their presence in clonal NMZL‐like B cells (Figure 2C). The *RHOA‐*G17V mutation was undetectable by Sanger sequencing, although its presence was confirmed by the more sensitive qPCR, suggesting a paucity of the neoplastic TFH cells in the microdissected sample. The *BRAF* mutation was not detected in PBMCs, cfDNA or, the early groin LN biopsy. The presence of both shared and distinct mutations in the AITL and the NMZL‐like proliferation indicates their divergent differentiation from a common progenitor (Figure 2D).

The patient was treated with six cycles of rituximab, cyclophosphamide, vincristine, doxorubicin and prednisolone (R‐CHOP), followed by rituximab maintenance therapy. Eight months post‐chemotherapy, PET‐CT showed that the lymphoma had a complete metabolic response (CMR).

### CASE 2: OCCULT NEOPLASTIC TFH CELL PROLIFERATION/SMOULDERING AITL OBSCURED BY A PREDOMINANT NEOPLASTIC CD8+ CTC PROLIFERATION

A 66‐year‐old male presented with generalized lymphadenopathy, an erythematous pruritic skin rash, and mild splenomegaly. No T‐large granular lymphocytosis was detected in peripheral blood. A right groin LN was excised and reported as a cytotoxic TCL, associated with an EBV+ large B‐cell proliferation. A skin biopsy was interpreted as reactive. The bone marrow (BM) trephine biopsy showed cytotoxic TCL, but an additional minor population of sCD3–/CD4+/CD10+ T cells, which was detected in the BM aspirate (0.75% of all CD45+ cells) by FCM (Table 1). Following CHOP chemotherapy, PET‐CT showed that the lymphoma had a CMR, but two separate new focal right upper and lower lobe lung lesions detected were diagnosed as synchronous primary adenocarcinoma and squamous cell carcinoma, respectively. Two months later, mediastinal lymphadenopathy identified on a CT scan prompted mediastinoscopic LN dissection in addition to resection of the lung tumours.

The mediastinal LNs showed nodal architecture effaced by a dense infiltrate of predominantly small to medium‐sized atypical lymphoid cells admixed with scattered large cells and clusters of epithelioid histiocytes. There was mild HEV hyperplasia. CD8+ cytotoxic T cells that showed down‐regulation of CD5 and CD7 predominated. In addition, there was a minor population of CD4+/CD10+/PD1+/ICOS+/CD8− atypical medium‐sized TFH cells that were concentrated around HEVs. Mildly hyperplastic CD21+ FDC meshworks surrounded HEVs. There were many individually scattered EBER+ large B cells (see Table 1 for a detailed immunoprofile). Clonality analyses identified a clonal T‐cell population, but no amplification of immunoglobulin gene targets. A diagnosis of an underlying smouldering AITL associated with a predominant neoplastic CTC proliferation/cytotoxic TCL and an expansion of EBV+ large cells was made.

**Figure 3:**
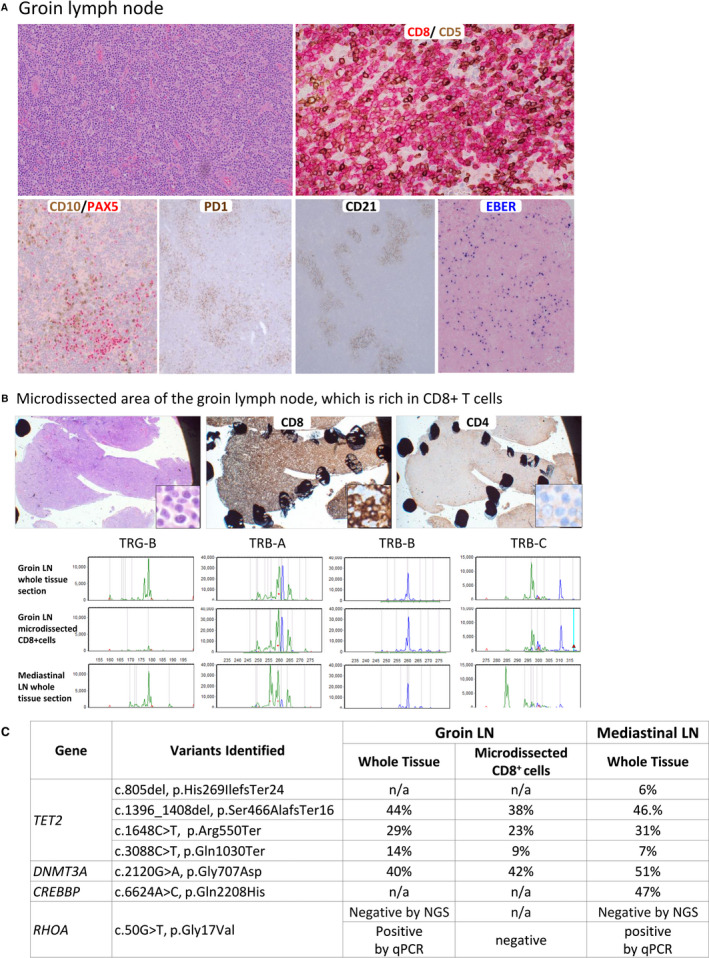
Histopathological and genetic findings in case 2. **A,** Upper panel: on haematoxylin and eosin staining (left), there is a dense diffuse monotonous infiltrate of small to medium‐sized lymphoid cells. CD5 (brown)/CD8 (red) double staining showed that most of the T cells expressed CD8, but had down‐regulation/loss of CD5 expression. Lower panels: CD10 (brown)/PAX5 (red) double staining (first panel) highlights a minor population of CD10‐positive, PAX5‐negative T cells that are positive for programmed cell death protein 1 (second panel). On CD21 staining (third panel) there is mild follicular dendritic cell hyperplasia, whereas, on Epstein–Barr virus (EBV)‐encoded small RNA *in‐situ* hybridisation (fourth panel) there is an increase in the number of EBV‐positive cells. **B,** Analysis of the CD8+ T cells of the groin lymph node (LN) by microdissection and BIOMED‐2 clonality assays. The CD8+ T‐cell population (microdissected from the tissue fragment showing extensive CD8 staining but not CD4 staining, marked by a dotted black line) is clearly clonal, showing a different clonal profile from those of whole tissue sections of both groin and mediastinal LNs, but a common clonal T‐cell receptor gene rearrangement by the TRB‐B reaction, thus being present prominently in both LN specimens. **C,** Summary of mutations detected in the two LN specimens and microdissected CD8+ T cells. CD8+ T cells harbour the *TET2* and *DNMT3A* mutations, but not the *RHOA*‐G17V mutation. The allelic frequencies of the variants included are based on Fluidigm PCR and MiSeq sequencing, and the sequencing coverage for all of the variant loci is adequate (mean of 657 reads and range of 196–1672 reads passing sequence quality checks). NA, not available.

The previous groin LN biopsy was reviewed, and was extensively necrotic; the viable areas were similar to the mediastinal LNs but lacked clusters of epithelioid histiocytes and contained more numerous EBV+ large B cells (Figure 3A; Table 1). Review of the BM biopsy confirmed the predominant CTC proliferation/cytotoxic TCL, but also identified a minor population of ICOS+/PD1 (moderate to strong)+/CD4+ cells that corresponded to the abnormal TFH cell population detected by FCM. Review of the skin biopsy showed subtle dermal infiltrates of neoplastic cytotoxic T cells, and CD4+/PD1 (strong)+/ICOS+/CD10+ TFH cells.

Clonality analysis, targeted sequencing and qPCR for the *RHOA*‐G17V mutation were performed on DNA samples from whole tissue sections of both the initial groin and subsequent mediastinal LN specimens, and from a small microdissected tissue area (of the groin LN) rich in CD8+ T cells but with very few CD4+ T cells (Figure 3B). Clonality analysis showed multiple clonal T‐cell receptor (TCR) gene rearrangements, with both common and distinct clonal peaks in whole tissue sections of both LNs indicating the presence of oligoclonal T‐cell populations (Figure 3B). The microdissected CD8+ cell‐rich population showed a different clonal profile from those of both the groin and mediastinal LNs, but shared a common clonal rearrangement detected by the TRB‐B reaction, indicating that the CD8+ cells were clonal and present prominently in both LN specimens.

NGS of the RMH lymphoma panel revealed multiple mutations in *TET2* (*n* = 4) and *DNMT3A* (*n* = 1), which were identical in both LN specimens and microdissected CD8+ T cells, with similar VAFs (Figure 3C; Figure S2A), and these changes were confirmed by Fluidigm PCR/Illumina MiSeq sequencing. Importantly, the *RHOA*‐G17V mutation, which was shown by qPCR to be present in whole tissue sections, was negative in the microdissected CD8+ T‐cell areas (Figure S2B), providing further reassurance that the population harbouring *TET2* and *DNMT3A* mutations in the latter were clonal CD8+ T cells. Targeted sequencing showed no evidence of *IDH2*, *STAT3*, *STAT5B* or *SETD2* mutation in either LN.

Following confirmation of lymphoma relapse, the patient was treated with rituximab, gemcitabine, dexamethasone and cisplatin, and remains in CMR 29 months after the first diagnosis.

## Discussion

These case studies show for the first time the parallel evolution of two distinct neoplastic lymphoid proliferations from a common haematopoietic progenitor cell population that carry *TET2* and *DNMT3A* mutations. These findings extend the previous reports of the development of metachronous AITL and myeloid neoplasms from a common *TET2*/*DNMT3A*‐mutated stem cell population in patients with clonal haematopoiesis.^9^, ^10^ Unlike the scenario of metachronous AITL and myeloid neoplasms, the two distinct lymphoid neoplastic proliferations in each case presented here involved the same LNs, with both cell populations intermingled together, making their identification highly challenging. In addition, as both lymphoid proliferations are within the same microenvironment, their clonal expansion and histological presentation are likely to impact on each other, and also be affected by the underlying inflammatory responses, particularly in the setting of clonal haematopoiesis.The extent of TFH cell proliferation in the two cases varies considerably, and a definitive conclusion regarding their malignant status is a challenge. In case 1, the groin LN biopsy showed extrafollicular FDC hyperplasia and a preferential distribution of TFH cells around hyperplastic HEVs in the absence of a significant B‐cell infiltrate, fulfilling the histological and immunophenotypic criteria for AITL. However, it was not possible to demonstrate clonal TCR gene rearrangement, owing to poor DNA quality. The *RHOA*‐G17V mutation was detected by qPCR, but its presence was inconclusive by Fluidigm PCR/MiSeq and it was not detected by Sanger sequencing, indicating that the mutation was present at a low allelic frequency. It is possible that the allelic frequency of the *RHOA*‐G17V mutation estimated by these PCR‐based assays is not entirely representative, owing to poor quality of DNA and the use of crude DNA preparations, resulting in a failure to demonstrate its true burden, which might match the extent of TFH cell proliferation shown by histopathological and immunophenotypic investigations. Alternatively, the *RHOA*‐G17V mutation might be a subclonal change or involve an independent clone, the TFH cell proliferation identified by histopathological and immunophenotypic investigations being driven by as yet unidentified genetic changes.

In case 2, the systemic dissemination of occult *RHOA*‐G17V‐mutant TFH cells and their preferential distribution around HEVs surrounded by mild extrafollicular FDC hyperplasia in LNs would be compatible with AITL. However, the occult nature of the TFH cell proliferation in both LN specimens and the predominant clonal CTC proliferation/lymphoma at relapse suggest that the TFH cell proliferation might represent a smouldering AITL, or an early manifestation of AITL.^20^

The impact of *TET2* and *DNMT3A* mutations on the survival and expansion of B cells and T cells is unclear. Individuals with clonal haematopoiesis do not usually show peripheral blood lymphocytosis.^21^ They may have circulating B cells and T cells harbouring *TET2* and *DNMT3A* mutations, but their allelic burden is far less than those seen in patients with AITL/TCL‐TFH, as exemplified by our cases. The high allelic burden of *TET2* and *DNMT3A* mutations in PBMCs of case 1, and in LN specimens of both cases, but in the absence of a predominant *RHOA*‐mutant TFH cell population, indicate their origin primarily from an NMZL‐like (case 1) or a CTC proliferation (case 2), and non‐neoplastic B cells and T cells descended from *TET2*/*DNMT3A*‐mutated haematopoietic stem cells. Thus, these non‐TFH cell proliferations are the primary culprits responsible for lymphadenopathy.

As the neoplastic TFH cells preserve at least some T‐helper functions, their clonal expansion and particularly their ectopic spatial extension may trigger dysregulated ‘exaggerated’ help to a range of immune responses through both cognate interactions and the release of soluble cytokines and ligands, such as CXCL13, CD40L, ICOS, interleukin (IL)‐6 and IL‐21, contributing to the polymorphous infiltrate commonly seen in tissue biopsies, particularly in patients with AITL associated with clonal haematopoiesis.^14^, ^15^, ^22^, ^23^ These have been elegantly demonstrated in *RhoA*‐G17V transgenic mice studies, in which CD4+ T cells expressing RhoA‐G17V are hyper‐reactive, drive marked autoimmunity, and result in histological features similar to those seen in human AITL.^14^, ^15^

In case 1, the development of an NMZL‐like proliferation may be promoted by both acquisition of *BRAF* mutations and active signalling through immune receptors, including those involved in T‐cell help, such as CD40, CD80/86, and cytokine receptors. It is of note that recurrent *BRAF* activation mutations have been reported in NMZL,^19^ and that the *BRAF*‐G469R mutation is a gain‐of‐function change, conferring intermediate BRAF kinase activity.^24^ In case 2, the clonal CTC proliferation/cytotoxic TCL may represent an extreme presentation of a deregulated immune response, potentiated by EBV reactivation and putative T‐cell help. In line with this, Pritchett *et al*. investigated the microenvironment of AITL, and demonstrated the expansion of CD8+ T‐cell populations with an exhausted phenotype, and an expression profile indicative of dysfunction and impaired cytotoxicity.^25^

The diagnosis and classification of these synchronous neoplastic lymphoid proliferations remain contentious with regard to whether they should be designated as secondary clonal lymphoid proliferations or secondary neoplasms. In case 1, the detection of both *TET2* and *BRAF* mutations may indicate some similarity with *de‐novo* NMZL.^26^ The relatively indolent clinical course, which would be unusual for AITL/TCL‐TFH, albeit it has been reported previously,^3^ taken in the context of a clonal, EBV–, *BRAF*‐mutant NMZL‐like proliferation, added to the diagnostic difficulty.

In case 2, despite the lack of distinct mutations in the CTC proliferation, down‐regulation of CD5 and CD7 is an aberrant phenotype. The mediastinal LN contained clusters of epithelioid histiocytes, which may potentially lead to a misdiagnosis of lymphoepithelioid/Lennert lymphoma, owing to the predominance of small to medium‐sized CD8+ T cells.^27^, ^28^ There is a well‐recognized morphological overlap between AITL and Lennert lymphoma,^28^, ^29^ and the diagnostic challenges in our case raise the possibility that some cases classified as the latter may represent AITL/TCL‐TFH associated with a predominant clonal CTC proliferation.

Although there have been occasional reports of AITL with a cytotoxic phenotype,^30^ the possibility of a florid CD8+ CTC proliferation obscuring a neoplastic TFH cell population was not explored. To the best of our knowledge, this is the first report of a predominant clonal CTC proliferation with an aberrant phenotype occurring in the context of an occult neoplastic TFH cell proliferation/smouldering AITL.

In summary, these cases highlight the need for close scrutiny, not only in the better‐known scenarios of EBV+/− Hodgkin/Reed Sternberg‐like cells and clonal proliferations of large B cells and plasma cells, but also in the setting of clonal EBV– small B‐cell/lymphoplasmacytic and CTC proliferations with prominent TFH cells, to exclude an underlying AITL/TCL‐TFH. Biologically, these cases studies expand the potential of clonal haematopoiesis in development of different lineage neoplastic proliferations.

## Conflicts of interest

The authors have no conflict of interest to declare.

## Author contributions

A. D. Attygalle revised the diagnoses. A. D. Attygalle and M.‐Q. Du analysed the data and co‐wrote the paper. R. Dobson performed and analysed the majority of the molecular tests and revised the manuscript critically, and Z. Chen processed the Fluidigm/NGS sequence data. D. Wren, H. Mugalaasi and Y Morgan performed the targeted NGS and clonality analysis, analysed the results of these, and revised the manuscript critically. P. K. Chak, K. M. Vroobel and K. N. Naresh provided histopathology input, analysed the data, and revised the manuscript critically. M. Kaur and R. Ahmad analysed the data and revised the manuscript critically.

## Supporting information


**Figure S1.** Examples of mutations detected by Fluidigm PCR/Illumina MiSeq sequencing in case 1.

**Figure S2.** Examples of mutations detected in case 2.

**Table S1.** Antibodies and conditions used for immunohistochemistry with the Ventana Benchmark Ultra IHC/ISH system.
**Table S2.** List of the genes included in the RMH lymphoma panel (197 genes).
**Table S3.** Primers used for Fluidigm or conventional PCR.

**Appendix S1.** Supplementary methods.

## Data Availability

All the data that support the findings of this study are presented in the main manuscript and supplementary material of this article.
