# Supplementary figures and images for: Parallel evolution of two distinct lymphoid proliferations in clonal haematopoiesis
Source: Histopathology. 2022 Mar 1;80(5):847–58. doi: 10.1111/his.14619 (PMC9310594; doi:10.1111/his.14619)

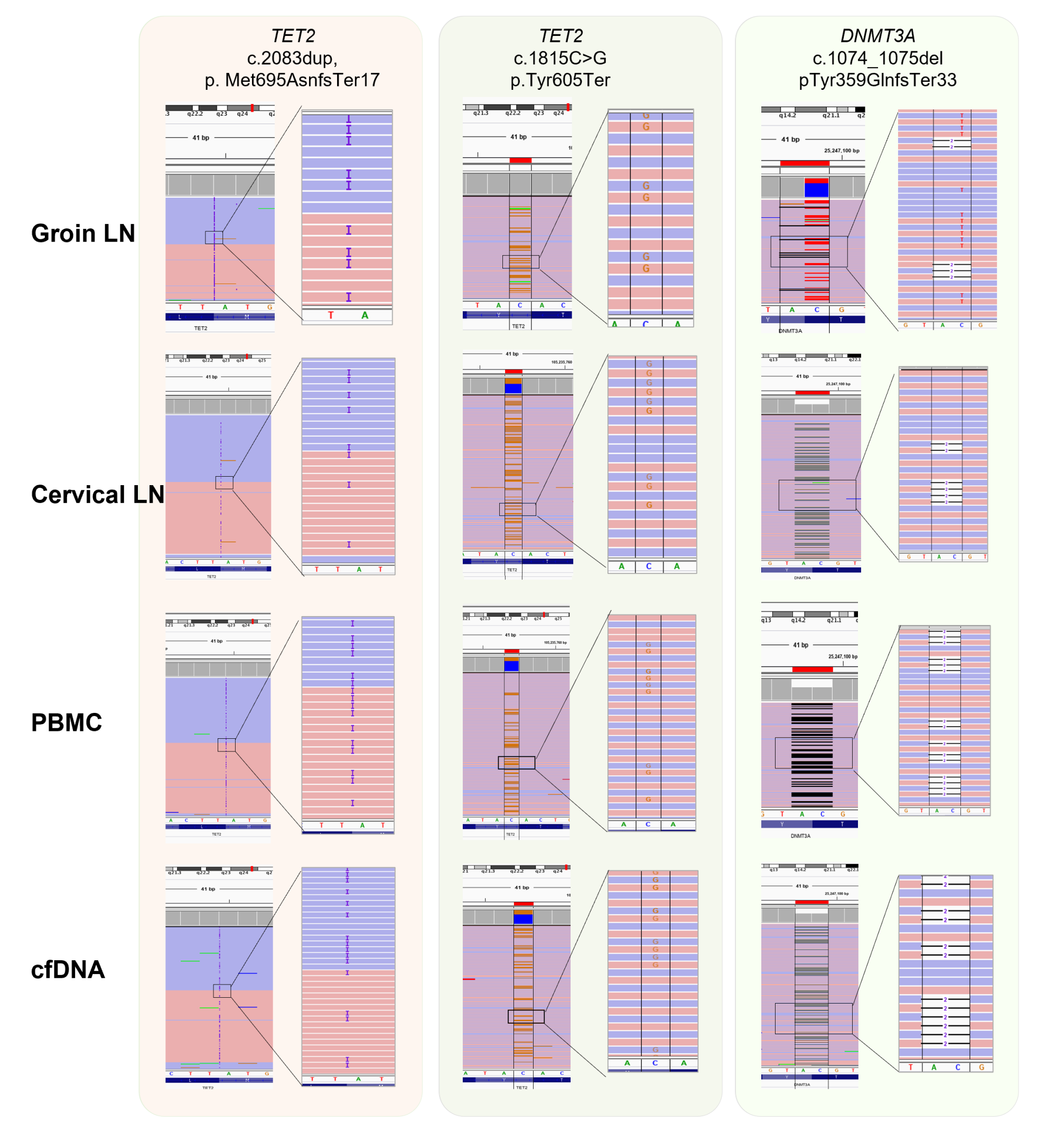

Supplement: Supplementary file 1 — Figure S1. Examples of mutations detected by Fluidigm PCR/Illumina MiSeq sequencing in case 1. [file HIS-80-847-s001.tif]

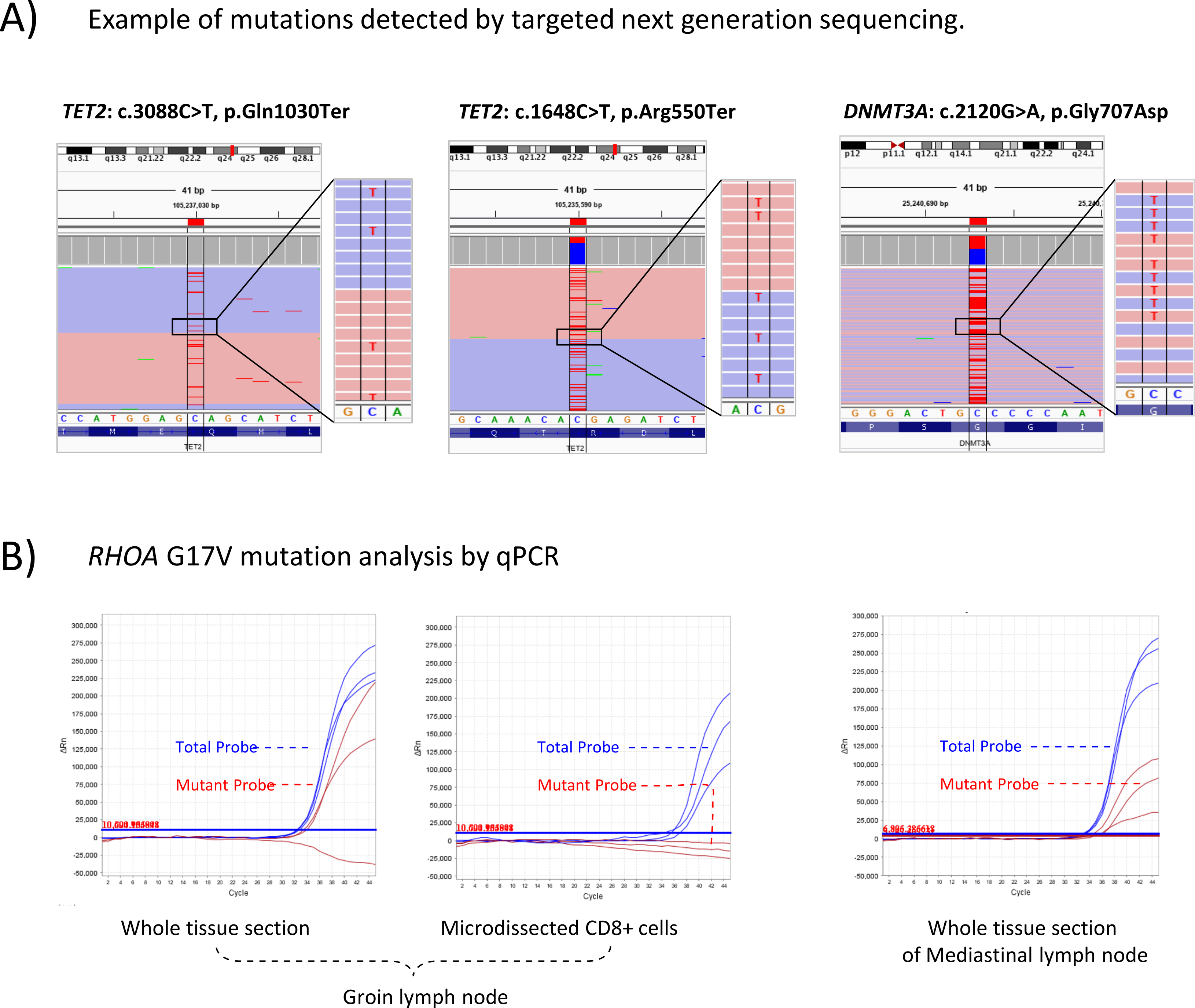

Supplement: Supplementary file 2 — Figure S2. Examples of mutations detected in case 2. [file HIS-80-847-s003.tif]
