# Supplementary material for: Parallel evolution of two distinct lymphoid proliferations in clonal haematopoiesis
Source: Histopathology. 2022 Mar 1;80(5):847–58. doi: 10.1111/his.14619 (PMC9310594; doi:10.1111/his.14619)
Supplement: Supplementary file 3 — Table S1. Antibodies and conditions used for immunohistochemistry with the Ventana Benchmark Ultra IHC/ISH system. Table S2. List of the genes included in the RMH lymphoma panel (197 genes). Table S3. Primers used for Fluidigm or conventional PCR. [file HIS-80-847-s002.docx]

**Supplementary Table S1:** Antibodies and conditions used for immunohistochemistry VENTANA BENCHMARK ULTRA IHC/ ISH SYSTEM

| **Antibody** | **Clone** | **Dilution** | **Manufacturer** | **Incubation time**  **(minutes)** |
| --- | --- | --- | --- | --- |
| **VENTANA BENCHMARK ULTRA IHC/ISH SYSTEM** | | | | |
| CD2 | MRQ-11 | RTU | Roche/Cell Marque | 32 |
| CD3 | LN10 | 1/50 | Leica | 32 |
| CD4 | SP35 | RTU | Roche/CONFIRM | 16 |
| CD5 | SP19 | RTU | Roche/CONFIRM | 16 |
| CD7 | CBC.37 | 1/20 | Agilent | 16 |
| CD8 | C8/144B | 1/25 | Agilent | 48 |
| BCL6 | LN22 | 1/50 | Leica | 60 |
| CD10 | 56C6 | 1/20 | Leica | 36 |
| PD1 | EPR4877(2) | 1/50 | Abcam | 48 |
| ICOS | SP98 | 1/25 | Abcam | 60 |
| TCR beta | 8A3 | 1/50 | Thermo Scientific | 60 |
| TIA1 | TIA1 | 1/25 | Abcam | 36 |
| Granzyme B | 11F1 | 1/30 | Leica | 32 |
| CD20 | L26 | 1/400 | Agilent | 16 |
| CD79A | JBC117 | 1/50 | Agilent | 32 |
| CD19 | BT51E | 1/25 | Leica | 44 |
| BCL6 | LN22 | 1/50 | Leica | 60 |
| cyclinD1 | SP4 | 1/40 | Menarini | 16 |
| IgD | ANTI HUMAN IgD | 1/1K | Agilent | 60 |
| CD23 | 1B12 | 1/20 | Leica | 60 |
| CD56 | 123C3 | RTU | Roche/CONFIRM | 28 |
| Kappa | KAPPA | 1/7.5K | Agilent | 40 |
| Lambda | LAMBDA | 1/7.5K | Agilent | 48 |
| EBER | INFORM EBER Probe with ISH iVIEW Blue Detection Kit | RTU | Roche | Protease:12  Probe: 16 |
| **LEICA BOND III** | | | | |
| CXCL13 | 53610 | 1/100 |  | 15 |
| CD57 | NK1 | 1/25 |  | 15 |
| CD10 (double stain) | 56C6 | 1/40 |  | 15 |
| PAX5 (double stain) | 24 | 1/50 |  | 15 |
| CD4 (double stain) | 4B12 | RTU |  | 15 |
| CD7 (double stain) | LP15 | RTU |  | 15 |
| CD5 (double stain) | 4C7 | 1/100 |  | 15 |
| PD1 (double stain) | NAT105 | 1/25 |  | 15 |
| CD8 (double stain) | 4B11 | RTU |  | 15 |

RTU: ready to use

**Supplementary Table S2:** List of the genes included in the RMH lymphoma panel (197 genes).

| ACVR1 | CDKN2A | FOXO1 | MSH6 | RHOA |
| --- | --- | --- | --- | --- |
| AKT1 | CDKN2B | GATA1 | MTOR | RICTOR |
| AKT2 | CDKN2C | GATA3 | MYC | RNF43 |
| ALK | CEBPA | GNA11 | MYCL | ROS1 |
| AMER1 | CHEK2 | GNAQ | MYCN | RUNX1 |
| ANTRX2 | CIC | GNAS | MYD88 | SETBP1 |
| APC | CKS1B | H3F3A | NF1 | SETD2 |
| ARAF | CREBBP | H3F3B | NF2 | SF3B1 |
| ARID1A | CRLF1 | HIST1H3B | NFE2 | SH2B3 |
| ASXL1 | CSF3R | HIST1H3C | NOTCH1 | SMAD2 |
| ATM | CTNNB1 | HIST2H3A | NOTCH2 | SMAD3 |
| ATRX | CUX1 | HIST2H3C | NPM1 | SMAD4 |
| AURKA | CXCR4 | HRAS | NRAS | SMARCA4 |
| B2M | DAXX | ID3 | NSD1 | SMARCB1 |
| BAP1 | DDR2 | IDH1 | PALB2 | SMARCE1 |
| BCL2 | DDX3X | IDH2 | PAX5 | SMO |
| BCOR | DICER1 | IGF1R | PBRM1 | SOX2 |
| BIRC3 | DIS3 | IKZF1 | PDCD1LG2 | SRSF2 |
| BRAF | DNMT3A | IRF4 | PDGFRA | STAG2 |
| BRCA1 | DROSHA | JAK1 | PHOX2B | STAT3 |
| BRCA2 | EGFR | JAK2 | PIK3CA | STAT5B |
| BRIP1 | EP300 | JAK3 | PIK3CD | SUFU |
| BTG1 | ERBB2 | KDR | PIK3R1 | TCF3 |
| BTK | ERBB3 | KIT | PLCG2 | TERT |
| CALR | ESR1 | KLF2 | PMS1 | TET2 |
| CARD11 | ETNK1 | KMT2A | PMS2 | TFE3 |
| CASP8 | ETV6 | KMT2C | POLE | TG |
| CBL | EZH2 | KMT2D | POT1 | TP53 |
| CCND1 | F2R | KRAS | PRKAR1A | TP63 |
| CCND2 | FADD | MAP2K1 | PTCH1 | TSC1 |
| CCNE1 | FAM46C | MAP2K4 | PTCH2 | TSC2 |
| CCNE2 | FAT1 | MAP3K1 | PTEN | U2AF1 |
| CD274 | FBXW7 | MCL1 | PTPN11 | VHL |
| CD79B | FGF10 | MDM2 | RAD21 | WT1 |
| CDH1 | FGFR1 | MDM4 | RAD51C | YAP1 |
| CDK12 | FGFR2 | MEF2B | RAD51D | ZRSR2 |
| CDK2 | FGFR3 | MET | RAF1 |  |
| CDK4 | FGFR4 | MLH1 | RB1 |  |
| CDK6 | FLT3 | MPL | RET |  |

**Supplementary Table S3:** Primers used for Fluidigm or conventional PCR.

| **Gene** | **Exon** | **primer name** | **Sequence (5'-3')** | **Amplicon size (bp)** | **Case** |
| --- | --- | --- | --- | --- | --- |
| TET2 | 3 | TET2_3_12f | GCACTCTGAATGGTGGAGTTT | 185 | Case 2 |
| TET2 |  | TET2_3_12r | CACACAATTATTCTGAGGCCTTT |  |  |
| TET2 | 3 | TET2_3_14f | TGACTGTTCCATTGTGTTCTGAG | 215 | Case 2 |
| TET2 |  | TET2_3_14r | CAATTCAATCCATCCTGGTTTC |  |  |
| TET2 | 3 | TET2_3_16f | GTATCAACCCAATCTCTCCAATCA | 185 | Case 1 |
| TET2 |  | TET2_3_16r | ACTGGAGATGTTGGTCCACTGT |  |  |
| TET2 | 3 | TET2_3_18f | CATGTGCAGTCACTGTGTGG | 208 | Case 1 |
| TET2 |  | TET2_3_18r | TTTGAGGGAGATGTGAACTCTG |  |  |
| TET2 | 3 | TET2_3_26f | CCACCAGAAAACAAAACATGG | 176 | Case 2 |
| TET2 |  | TET2_3_26r | TTTACTTGCTTCTGTGATTTGAGA |  |  |
| DNMT3A | 9 | DNMT3A_9f | GCAAACAAGGCCTGGCTT | 210 | Case 1 |
| DNMT3A |  | DNMT3A_9r | CCGACCTGCACTCCAACT |  |  |
| DNMT3A | 18 | DNMT3A_18f | ACGTTGCCTTTATCCTCCCA | 209 | Case 2 |
| DNMT3A |  | DNMT3A_18r | CATCGGGAATAGCTGTCCCA |  |  |
| RHOA | 2 | RHOA_2_1f | AAGCTACCTATGACTTCTTGTGC | 205 | Cases 1 & 2 |
| RHOA |  | RHOA_2_1r | CATCCACCTCGATATCTGCCA |  |  |
| BRAF | 12 | BRAF_f | AACACTTGGTAGACGGGACTC | 135 | Case 1 |
| BRAF |  | BRAF_r | CACCACATTACATACTTACCATGCC |  |  |

The CS1 tag (ACACTGACGACATGGTTCTACA) was added to the 5' of the forward primer and the CS2 tag (TACGGTAGCAGAGACTTGGTCT) was added to the 5' of the reverse primer and used for barcoding.

Fluidigm PCR were carried out using 5ng DNA in duplicates using the following cycling parameters.

| **PCR Stages** | **Number of Cycles** |
| --- | --- |
| 50ºC for 2 mins | 1 |
| 70ºC for 20 mins | 1 |
| 95ºC for 10 mins | 1 |
| 95ºC for 15 s | 10 |
| 60ºC for 30 s |  |
| 72ºC for 1 min |  |
| 95ºC for 15 s | 2 |
| 80ºC for 30 s |  |
| 60ºC for 30 s |  |
| 72ºC for 1 min |  |
| 95ºC for 15 s | 8 |
| 60ºC for 30 s |  |
| 72º C for 1 min |  |
| 95ºC for 15 s | 2 |
| 80ºC for 30 s |  |
| 60ºC for 30 s |  |
| 72ºC for 1 min |  |
| 95ºC for 15 s | 8 |
| 60ºC for 30 s |  |
| 72ºC for 1 min |  |
| 95ºC for 15 s | 5 |
| 80ºC for 30 s |  |
| 60ºC for 30 s |  |
| 72ºC for 1 min |  |
