# Supplementary material for: Parallel evolution of two distinct lymphoid proliferations in clonal haematopoiesis
Source: Histopathology. 2022 Mar 1;80(5):847–58. doi: 10.1111/his.14619 (PMC9310594; doi:10.1111/his.14619)
Supplement: Supplementary file 4 — Appendix S1. Supplementary methods. [file HIS-80-847-s004.docx]

**Supplementary methods**

**Microdissection and DNA extraction**

Purified DNA samples were prepared from whole tissue sections of formalin fixed paraffin embedded tissues using QIAamp DNA-Micro kit (Qiagen). Where indicated, defined cell populations from consecutive sections were isolated by manual microdissection under the light microscope under 10×objective lens using a disposable Terumo Agani Needle. In case 1, areas enriched by the B-cell/plasmacytic NMZL-like infiltrate but lacking prominent PD1 positive cells in the cervical lymph node were first identified based on haematoxylin & eosin and PD1 staining, and corresponding areas on consecutive sections were microdissected as outlined above. In case 2, a tissue fragment (~20,000 cells) within the groin lymph node, which showed extensive CD8+, but not CD4+ T-cells was similarly microdissected. The microdissected cells from case 1 were then subjected to crude DNA preparation by digestion with proteinase-K.^1^ The groin LN biopsy from case 1 was also subjected to crude DNA preparation, due to limited tissue availability. These crude DNA preparations were used for PCR based analyses including PCR/Sanger sequencing, clonality analysis, Fluidigm PCR and Quantitative PCR with peptide nucleic acid clamp and locked nucleic acid probe. Meanwhile, purified DNA samples were prepared for microdissected cells from case 2. The quality of DNA samples was assessed by PCR of variably sized genomic fragments using a standardised protocol as previously described.^2^

**Targeted sequencing using RMH lymphoma panel (197 genes)**

A total of 1 µg DNA was hybridized overnight with a custom design of DNA baits complementary to the genomic regions of interest (Nimblegen, Roche Sequencing, Basel, Switzerland). Hybridised DNA was PCR amplified and products purified using AMPure XP beads (Beckman Coulter, Danvers, MA, USA) and quantified using the Qubit dsDNA High Sensitivity Assay Kit with the Qubit 3.0 fluorometer (Invitrogen, Carlsbad, CA), and High Sensitivity D1000 TapeStation (Agilent, Santa Clara, USA). Next generation sequencing (NGS) libraries were prepared using the KAPA HyperPlus Kit (Kapa Biosystems, Wilmington, MA, USA) and IDT UDI 8bp adapters (Integrated DNA Technologies, Coralville, USA), following the manufacturer’s protocol, including dual-SPRI size selection of the libraries (250-500 bp), then sequenced on a NovaSeq6000 (Illumina, San Diego, CA, USA) with 100bp paired end reads and v1 chemistry, according to the manufactures instructions.

The sequencing data were analyzed using an in-house variants calling pipeline. For demultiplexing, bcl2fastq (v2.19) was used to isolate reads for each sample. The reads were aligned to the reference genome build GRCh37/Hg19 using Burrows–Wheeler Aligner (BWA-MEM), followed by the marking of PCR duplicates and calculation of various QC metrics using Picard. Genome Analysis ToolKit (GATK) was used for realigning around indels to improve indel calling and base quality score recalibration for adjusting systematic errors made by the sequencer when estimating quality scores of each base call. GATK-Mutect was used for variant calling for tumour only analysis and tumour-normal paired analysis. Copy number variation (CNV) was assessed by measuring the GC adjusted coverage ratio between each tumour probe target and the average coverage of all probe targets in the sample. The coverage ratio is further normalised against pooled baseline ratio of over 100 samples. Finally, Manta (v.0.29.6) and Pindel were used for the detection of structural variants.

All potential mutations, structural variants, and CNVs were visualized using Integrative Genomics Viewer; the following filtering criteria were applied: minimum variant allele depth 10x, Variant Allele Frequency (VAF) threshold at 5%.

**Mutation analysis by Fluidigm PCR and Illumina MiSeq sequencing**

The variants idenfified by the above panel sequencing were validated by Fluidigm PCR and Illumina MiSeq sequencing as described previously.^3^ Briefly, the Fluidigm PCR was carried out with 5ng input DNA in duplicate using the FastSart high fidelity system (Roche) with appropriate primers with a common sequence (CS) tag, and the primer sequences, amplicon sizes and PCR conditions were detailed in Supplementary Table 3. PCR products were purified using AMPure XP beads and sequenced using Illumina Miseq. Sequence reads alignment, variant calling and filtering were carried out according to our previously established protocols.^1^

Where indicated, PCR products were also sequenced using the Sanger method from both directions. Sanger sequencing data were analyzed using Snapgene 5.2 software. All PCRs and sequencing were carried out in duplicates.

**Quantitative PCR with peptide nucleic acid clamp and locked nucleic acid probe (PNA-LNA qPCR)**

The qPCR assay contained two probes and a PNA clamp.^1^ The PNA clamp specifically and strongly binds to the wild type DNA sequence, and blocks the wild type allele from PCR amplification. This results in preferential amplification of the mutant allele which is detected specifically by the LNA mutant probe. The PNA-LNA PCR was carried out in a 20µl reaction containing 10µL Premix Ex taq (Probe qPCR) Master Mix (Takara, Shiga, Japan), 0.2µM each forward and reverse primer, 0.1µM each total and mutant probe, 0.05µM PNA clamp probe, and 2-7µL of crude (groin lymph node and microdissected NMZL-like cells from cervical lymph node in case 1) or 25ng purified DNA. Real time PCR was carried out in triplicate using Quantstudio 6 (Thermo Fisher Scientific, Waltham, MA, USA) with denaturation at 95°C for 30s followed by 45 cycles at 95°C for 3s, and 62°C for 30s.

**References:**

1. Dobson R, Du PY, Rásó-Barnett L *et al.* Early detection of T-cell lymphoma with T follicular helper phenotype by RHOA mutation analysis. *Haematologica.* 2021.

2. Cucco F, Clipson A, Kennedy H *et al.* Mutation screening using formalin-fixed paraffin-embedded tissues: a stratified approach according to DNA quality. *Lab Invest.* 2018; **98:** 1084-1092.

3. Yao WQ, Wu F, Zhang W *et al.* Angioimmunoblastic T-cell lymphoma contains multiple clonal T-cell populations derived from a common TET2 mutant progenitor cell. *J Pathol.* 2020; **250:** 346-357.
